# Supplementary material for: Hypothermia Shifts Neurodegeneration Phenotype in Neonatal Human Hypoxic–Ischemic Encephalopathy but Not in Related Piglet Models: Possible Relationship to Toxic Conformer and Intrinsically Disordered Prion-like Protein Accumulation
Source: Cells. 2025 Apr 12;14(8):586. doi: 10.3390/cells14080586 (PMC12025496; doi:10.3390/cells14080586)
Supplement: Supplementary file 1 [file cells-14-00586-s001.zip › cells-3557967-supplementary.pdf]

Supplementary Information

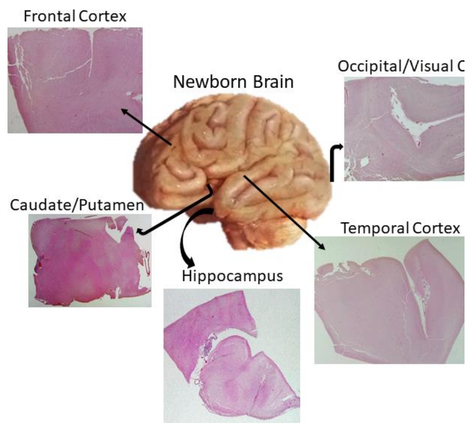

Figure S1. Regions in the human neonatal autopsy brain that were sampled for paraffin histology and analyzed.

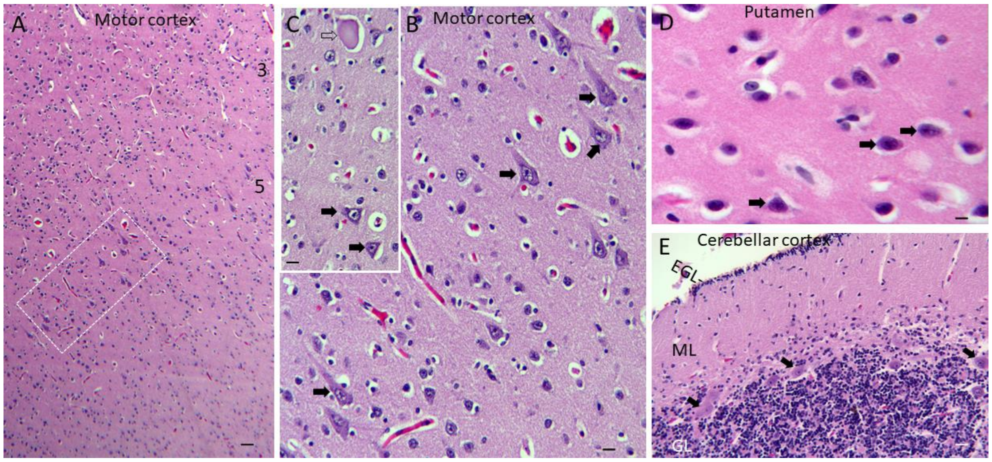

Figure S2. H&E staining of neocortex, basal ganglia, and cerebellum in a representative infant non-HIE case of SMA. (A) Motor cortex showing minimal tissue edema, relative clear lamination, and minimal cellular eosinophilia. Hatch white box is shown as panel B. Scale bar= 96 µm. (B) Layer 5 of motor cortex showing the Betz cells (arrows) and surrounding smaller neurons with no cytoplasmic eosinophilia, nuclear pyknosis, and minimal swelling or neuropil vacuolation. Scale bar= 37 µm. (C) Some layer 5 neurons showed a dramatic chromatolytic reaction with ballooning and cytoplasmic homogenization (open arrow), consistent with SMA and upper motor neuron changes, but nearby pyramidal neurons appeared normal (black arrows). Scale bar= 40 µm. (D) Putamen is generally free of classic ischemic neuropathology with most principal neurons appearing normal (arrows). Scale bar= 12 µm. (E). The cerebellar cortex is generally free of classic ischemic neuropathology with most Purkinje neurons appearing normal (arrows) as a layer between the molecular layer (ML) and granule cell layer (GL). The infantile status of this brain is indicated by the presence of the external granule cell layer (EGL). Scale bar= 20 µm.

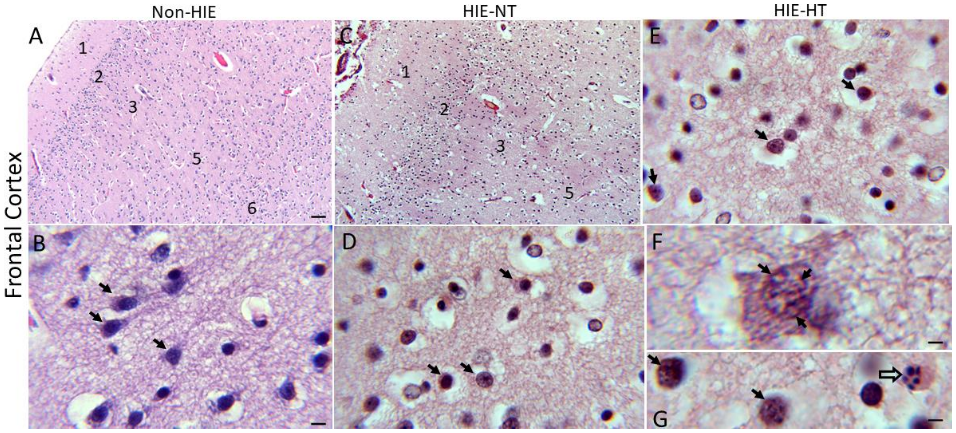

Figure S3. Neocortical neuropathology in human infant HIE. Representative H&E staining of paraffin sections of frontal cortex in cases of non-HIE (A and B), HIE without cooling (normothermia, NT, C and D), and HIE treated with hypothermia (HT, E, F, G). (A,B) In non-HIE cases, the neuropil is homogenously pink with layer discernment (1-6) and many interspersed intact neuronal cell bodies (B, arrows). Scale bars = 112 µm (A same for C); 22.5 µm (B, same for D, E). (C) In cases of HIE-NT, the frontal cortex neuropil becomes pale and edematous and layer discernment become equivocal. (D) In HIE-NT, the cortical neurons undergo cytotoxic edema with dissolution of the cytoplasm and cytoplasmic and nuclear eosinophilic pathology (arrows) and nuclear condensation. (E) The neuropathological changes seen in the frontal cortex of HIE-HT cases are generally similar to those seen in HIE-NT cases. (F,G) Occasionally in HIE-HT cases, degenerating cortical pyramidal neurons manifest with a cell death continuum phenotype seen at incipient nuclear clumping stages (F, arrows) and at endstage (G, open arrow) with neurotic neurons nearby (G, black arrows). Scale bars = 3 µm (F),10 µm (G).

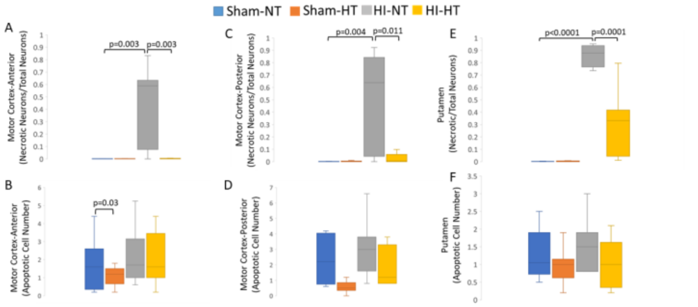

Figure S4. Piglets with hypoxia-ischemia (HI) and overnight hypothermia (HT) treatment have protection in the neocortex and putamen without a shift along the cell death continuum. (A,C,E) Piglets with HI and normothermic (NT) recovery have significant numbers of ischemic neurotic neurons in motor cortex at anterior (A) and posterior (C) neuroanatomical levels and in putamen (E) compared to sham-NT treatment with negligible ischemic-necrosis. HT significantly rescued the neurons in motor cortex and putamen from ischemic necrosis. HT was not deleterious in shams. (B,D,F) Hypothermia in HI piglets was protective in anterior and posterior motor cortex and in putamen without a corresponding increase in degeneration appearing as apoptotic or continuum. Sham-NT (n=6), sham-HT (n=10), HI-NT (n=8), and HI-HT (n=10). Statistically significant *P* values are indicated.

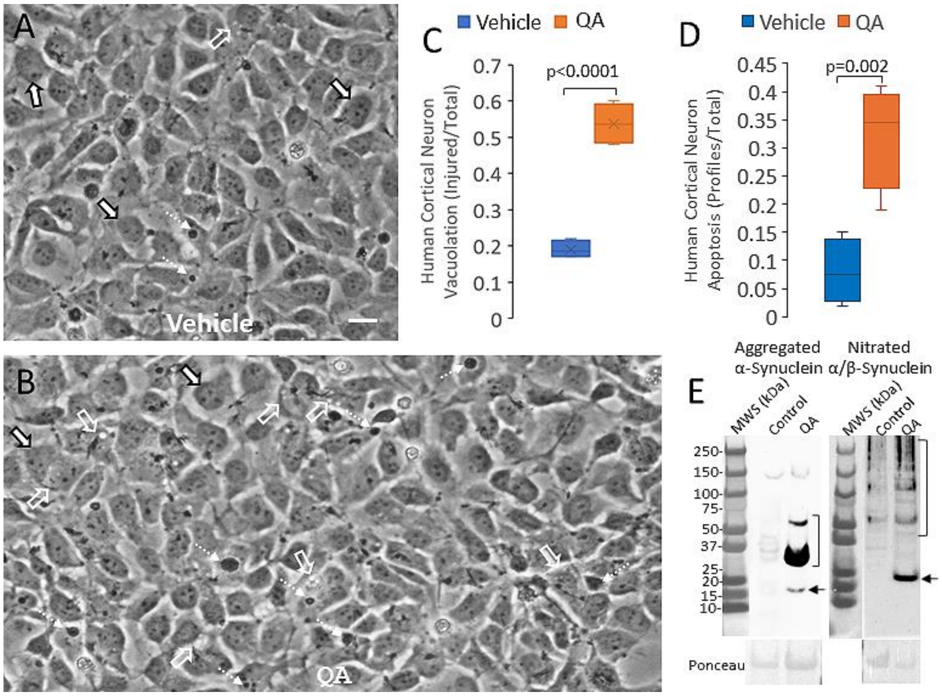

Figure S5. Human neuron model of excitotoxicity and toxic conformer formation using the H9 embryonic stem cell line differentiated to neurons. (A,B) Live cell images showing vehicle treated (A) and quinolinic acid (B, QA) treated neurons. The neurons are seen as normal (solid white broad arrows), vacuolated neurons (open white broad arrows), and apoptotic (hatched thin white arrows). (C,D) Quantification of excitotoxic damage in human neurons seen as cells with vacuolar pathology (C) or apoptotic morphology (D). Box plots show mean values (with IQR and 5-95 percentile whiskers. Three independent experiments were done. Significant *p* values are identified. (E) Western blots showing accumulation aggregated α-Syn (left) and nitrated-Syn (right) in QA treated human neurons. Black arrows identify monomers. Brackets identify putative aggregate forms. Ponceau stained membranes (bottom) show protein loading.

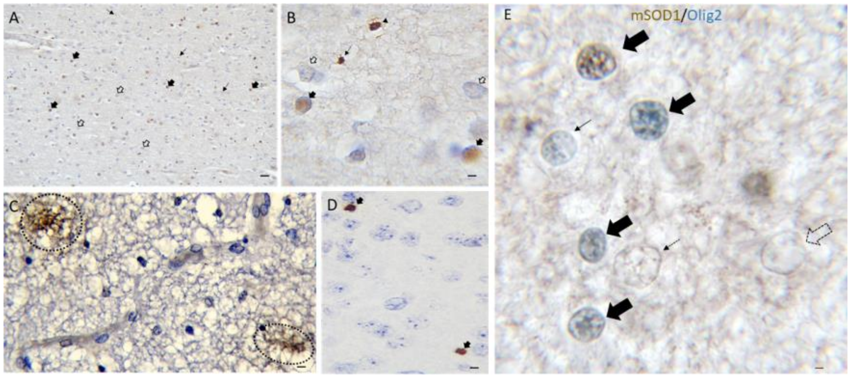

Figure S6. Misfolded/oxidized SOD1 accumulates in white matter cells of human HIE cases. (A,B) Forebrain subcortical white matter contains numerous cells that are positive for misfolded/oxidized SOD1 (A, black arrows) and appeared to be oligodendrocytes based on nuclear morphology (B, black arrows) and confirmed as oligodendrocytes by immunophenotyping (E). Other nearby cells are not positive (A and B, open arrows). Numerous small misfolded/oxidized SOD1-positive profiles, possibly apoptotic debris or endstage cells (A and B, thing arrows), are also present in white matter. Scale bars = 21 µm (A),12 µm (B). (C) In cerebellar white matter, diffuse plaques (hatched ellipses) were positive for misfolded/oxidized SOD1. Scale bar = 10.5 µm. (D) Misfolded/oxidized SOD1-positive smalls cells (black arrows) were present in hippocampal white matter. Scale bar = 7 µm. (E) Colocalization of aberrant SOD1 (brown) in Olig2-positive (blue-gray) oligodendrocytes in subcortical white matter. SOD1 single labeling (thin hatched arrow). Olig2 single labeling (solid black thin arrow). Double labeled cells (solid black broad arrows). A-D are single labeling counterstained with CV. E is not counterstained with CV. Scale bar = 4 µm.
