# Supplementary figures and images for: Hypothermia Shifts Neurodegeneration Phenotype in Neonatal Human Hypoxic–Ischemic Encephalopathy but Not in Related Piglet Models: Possible Relationship to Toxic Conformer and Intrinsically Disordered Prion-like Protein Accumulation
Source: Cells. 2025 Apr 12;14(8):586. doi: 10.3390/cells14080586 (PMC12025496; doi:10.3390/cells14080586)

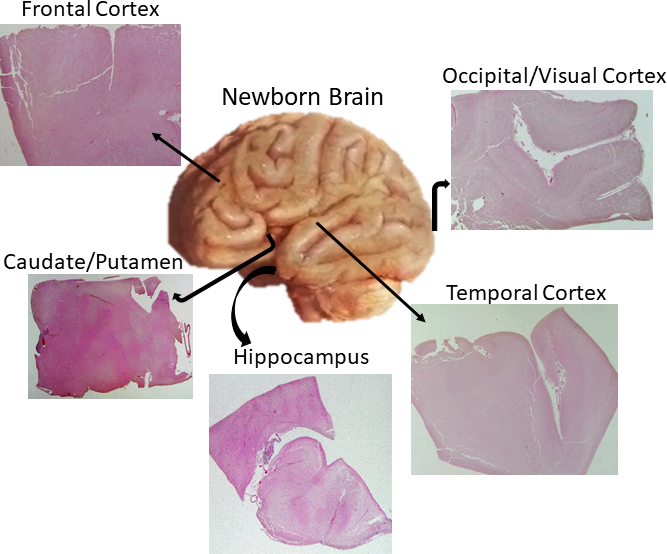

Supplement: Supplementary file 1 [file cells-14-00586-s001.zip › Figure S1.tif]

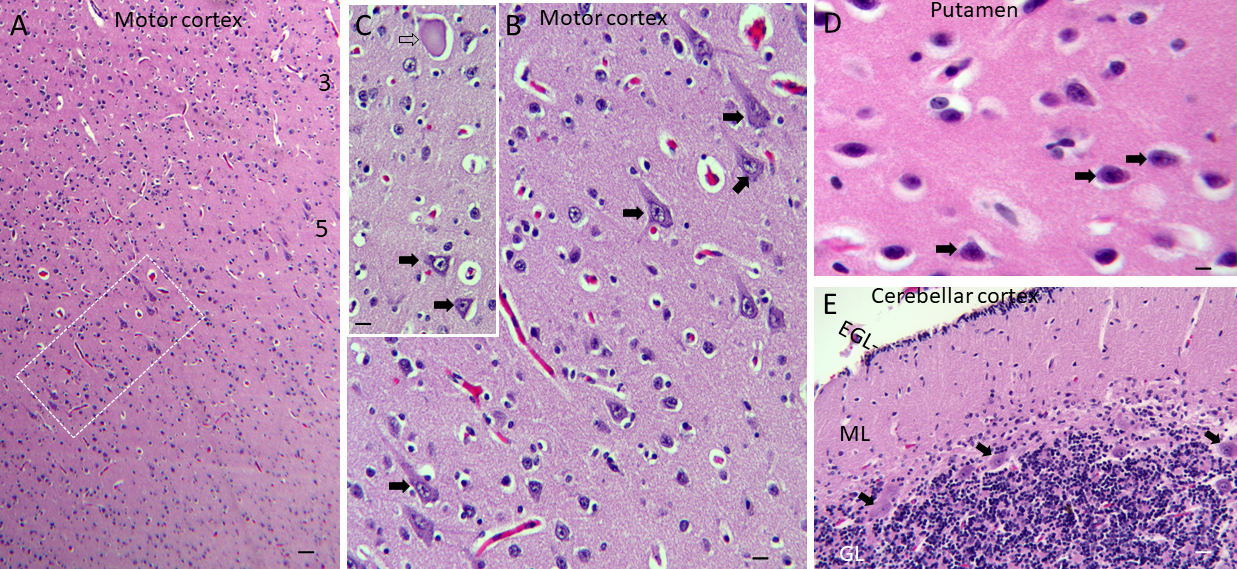

Supplement: Supplementary file 1 [file cells-14-00586-s001.zip › Figure S2.tif]

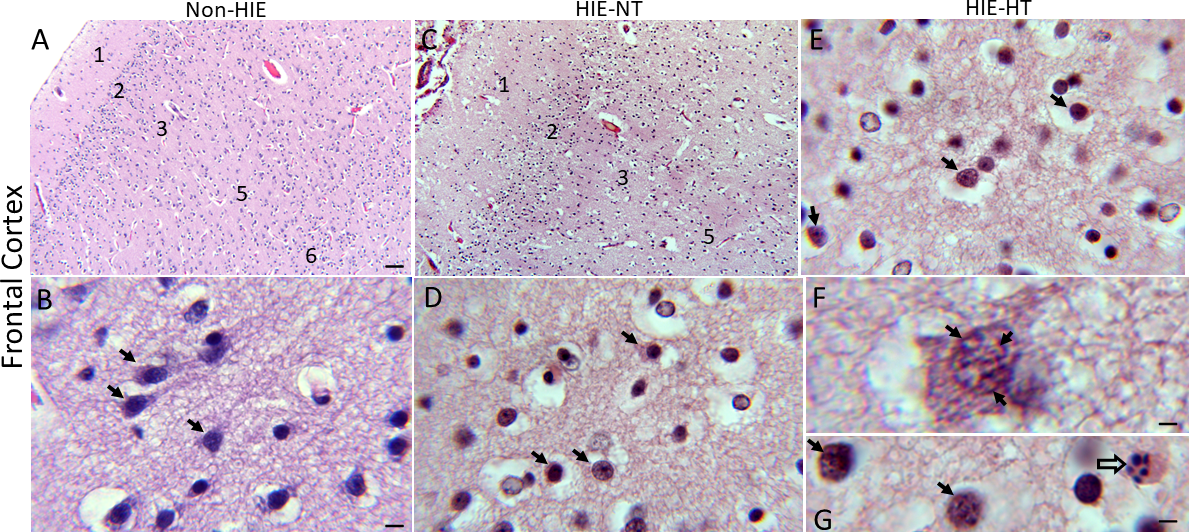

Supplement: Supplementary file 1 [file cells-14-00586-s001.zip › Figure S3.tif]

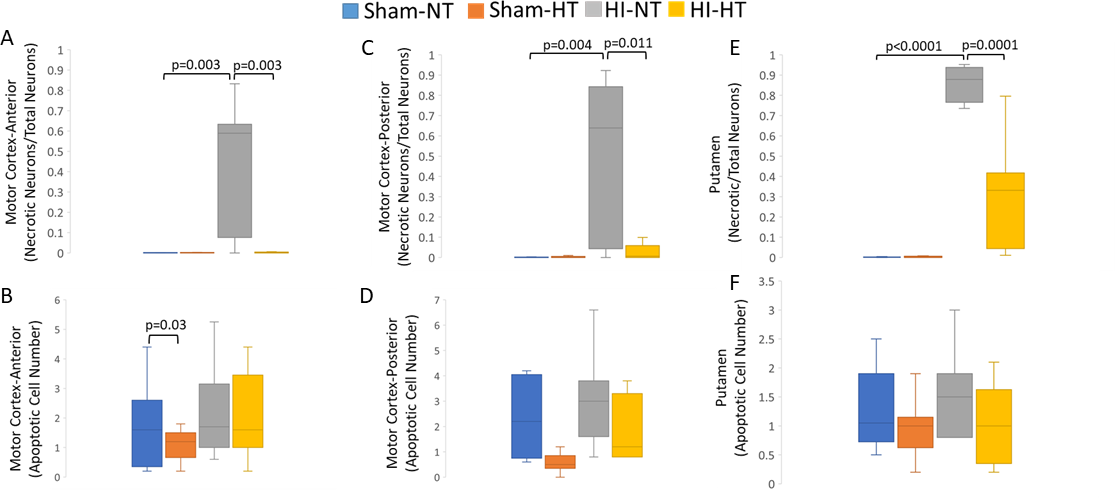

Supplement: Supplementary file 1 [file cells-14-00586-s001.zip › Figure S4.tif]

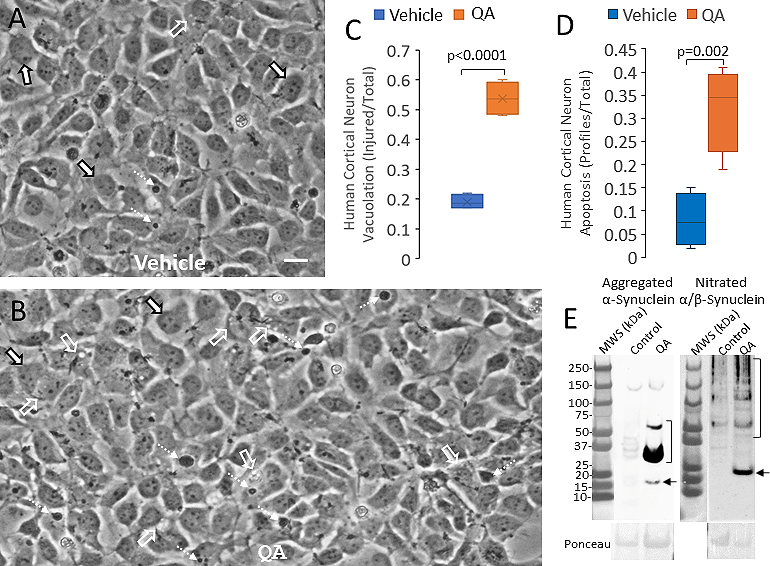

Supplement: Supplementary file 1 [file cells-14-00586-s001.zip › Figure S5.tif]

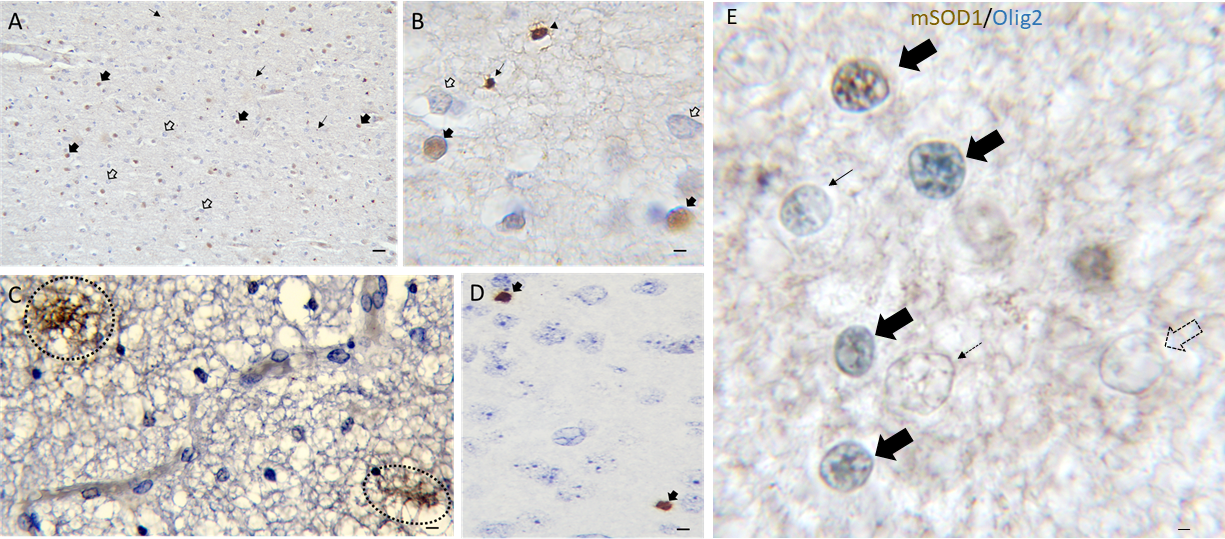

Supplement: Supplementary file 1 [file cells-14-00586-s001.zip › Figure S6.tif]
